# Studying Suicidality Among Psychiatric Nurses in Tunisia

**DOI:** 10.1192/j.eurpsy.2026.10696

**Published:** 2026-07-31

**Authors:** M. Bouchendira, A. Maamri, A. Amara, A. Hajri, A. Karmous, H. Zalila

**Affiliations:** 1 Emergencies and consultations, RAZI hospital, Manouba; 2 Community and Preventive Medicine, Faculty of medicine of Sousse, Sousse, Tunisia

## Abstract

**Introduction:**

Psychiatric nurses are frequently exposed to emotionally demanding and potentially traumatic events, including workplace aggression and patient suicide attempts. These experiences can increase psychological distress and elevate the risk of suicidality. Understanding the role of coping strategies in this context is essential for designing effective preventive interventions.

**Objectives:**

To assess the prevalence of suicidality among psychiatric nurses and examine its association with workplace trauma, coping strategies and psychological distress

**Methods:**

An exhaustive cross-sectional study was conducted among 237 psychiatric nurses in every public psychiatric unit in Tunisia. Data were collected using the Suicidal Behaviors Questionnaire-Revised (SBQ-R) to assess suicidality, the Brief COPE inventory to identify coping strategies, The Depression Anxiety Stress Scale (DASS-21) to measure psychological distress, Exposure to workplace aggression and witnessing patient suicide attempts was also documented through interviews.

**Results:**

Among the 237 participants, 4.2% were identified as high risk for suicidality according to SBQ-R scores. Exposure to workplace aggression was reported by 67.5% of nurses, while 45.1% had witnessed a patient suicide attempt. On the DASS-21, 28.3% of participants had moderate to severe depression, 24.9% had moderate to severe anxiety, and 31.2% had moderate to severe stress. Suicidality scores were positively correlated with DASS-21 subscale scores, with higher suicidality observed among participants reporting elevated depression, anxiety, and stress. Regarding coping strategies, 38.4% predominantly used avoidant coping (e.g., behavioral disengagement, denial), whereas 61.6% reported frequent use of problem-focused strategies (e.g., planning, active coping). Both witnessing patient suicide attempts and experiencing workplace aggression were associated with higher SBQ-R scores (p < 0.05). Suicidality scores were higher among participants using avoidant coping strategies (p = 0.002), while lower scores were observed in those using problem-focused strategies.

**Conclusions:**

Suicidality among psychiatric nurses is strongly linked to workplace trauma and maladaptive coping, whereas adaptive coping strategies appear protective. These findings underscore the importance of institutional support and training in adaptive coping skills to safeguard nurses’ mental health. Prospective studies are needed to confirm causal relationships and guide preventive interventions.

**Disclosure of Interest:**

None Declared

